# Bilateral Prophylactic and Contralateral Risk‐Reducing Mastectomy in Women With Germline *CDH1* Pathogenic or Likely‐Pathogenic Variants

**DOI:** 10.1002/jso.70323

**Published:** 2026-07-12

**Authors:** Giovanni Corso, Carlo La Vecchia, Francesca Magnoni, Andrea Polizzi, Elisa Bottazzoli, Alessandra De Scalzi, Susanna Di Silvestre, Eleonora Meduri, Filippo Pesapane, Luca Nicosia, Francesca De Lorenzi, Mattia Intra, Viviana Galimberti, Paolo Veronesi

**Affiliations:** ^1^ Division of Breast Surgery European Institute of Oncology, IRCCS Milan Italy; ^2^ Department of Oncology and Hemato‐Oncology University of Milan Milan Italy; ^3^ Department of Clinical Sciences and Community Health University of Milan Milan Italy; ^4^ Division of Breast Radiology European Institute of Oncology, IRCCS Milan Italy; ^5^ Division of Plastic and Reconstructive Surgery European Institute of Oncology, IRCCS Milan Italy

## Abstract

Bilateral prophylactic mastectomy (BPM) or contralateral risk‐reducing mastectomy (CRRM) are considered in *CDH1* carriers as a strategy to reduce lobular breast cancer (LBC) risk in women. In this study we aimed to evaluate the overall number of these procedures reported in literature. We conducted a literature search and a scoping review through the PUBMED database. We identified eleven studies eligible for our interest. Overall numbers, frequencies, geographic distribution, family history, and post‐operative histopathological findings of BPM or CRRM were the main outcomes considered. Articles reporting information about BPM or CRRM were reported only in Europe and North America. A total of 75 (32.1%) women were treated with any mastectomy (BPM or CRRM), from a population of 234 positive at *CDH1* genetic testing; 81.3% of these women, were treated in North America and 18.7% in Europe. In total, 69.3% of surgical procedures were BPM and 30.7% were CRRM; 76.9% of BPMs were performed in North America, and 23.1% in Europe. Instead, 91.3% of CRRMs were reported in North America, and 8.7% in Europe, respectively. A positive family history for BC was documented in 81.8% and post‐operative histopathological findings described at least one in situ LBC in nearly all breast specimens. In conclusion, BPM or CRRM were performed in about 1/3 of women with germline *CDH1* pathogenic or likely pathogenic variants; studies were reported only in Western countries. Family history for BC in *CDH1* carriers and detection of LCIS in post‐operative specimens were also frequently described

## 1 Introduction

Germline *CDH1* pathogenic or likely‐pathogenic variants confer an increased risk to develop lobular breast cancer (LBC) and/or diffuse gastric cancer (DGC) [1], within the well‐known inherited disease called hereditary diffuse gastric and lobular breast cancer syndrome. However, the risk and the frequency are very different between lobular and gastric tumors. The hereditary DGC (HDGC) [2] is an inherited cancer predisposition syndrome associated prevalently with DGC risk; however, germline *CDH1* pathogenic or likely‐pathogenic variants are also identified in women with a first manifestation of LBC, but not obligately with a DGC phenotype. This new rare predisposition was called hereditary lobular breast cancer syndrome (HLBC) [3, 4, 5]. In 1998, the first description of germline *CDH1* variant was reported in Māori kindred with a strong aggregation for DGC and LBC [2]. Successive large population screening revealed that this syndrome is a rare predisposition disease, accounting for about 3% as cases of familial GC and 1% as hereditary setting [6].

The cumulative lifetime GC risk at age 80 is variable according to family history for GC and it is estimated around 70%–40% for males and 56%–25% for females [7, 8, 9]. A recent report documents a much lower cumulative risk for GC with a cumulative risk of advanced GC by age 80 years of 10% for male and 6.5% for female *CDH1* carriers, in a sporadic setting [10]. The lifetime risk of LBC for women with *CDH1* mutations ranges from 37% to 55%, depending on the study population [11]. While we are seeing a decrease in risk for DGC, it does not appear to be the case for LBC. Ryan et al., reported in 2024 a stable trend of LBC risk in women with germline *CDH1* pathogenic or likely pathogenic variants (around 37%), independently of the BC family history [10].

Due to the well‐documented risk of DGC and LBC in *CDH1* carriers, prophylactic total gastrectomy (PTG) and bilateral prophylactic mastectomy (BPM) or contralateral risk‐reducing mastectomy (CRRM), for those women receiving prior diagnosis of LBC, are considered a possible procedure to mitigate the risk in *CDH1* carriers. However, very few BPM or CRRM procedures are reported in literature, mostly described as case reports.

This study aimed to review literature to evaluate the frequency of BC risk‐reducing surgical procedure in *CDH1* carriers.

## 2 Methods

**Figure 1:**
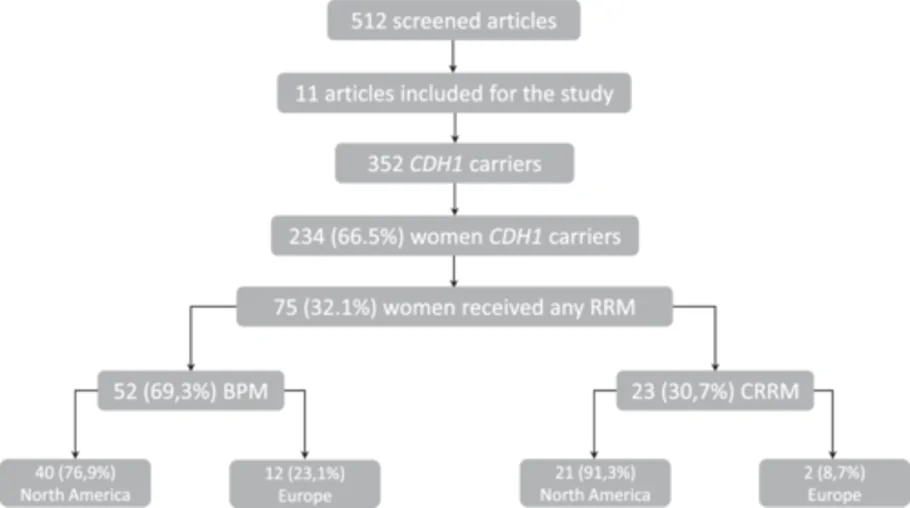
Flow chart of studies selection and description of the main results. Abbreviations: RRM, risk‐reducing mastectomy; BPM, bilateral prophylactic mastectomy; CRRM, contralateral risk‐reducing mastectomy.

To identify articles of our interest in this scoping review, we revised all cases of BPM or CRRM identified in *CDH1* carriers reported in MEDLINE (PubMed) from 1977 to 2025, including original reports and literature reviews edited in English (Figure 1). The analysis was limited to studies involving subjects treated with risk‐reducing surgery identified in these specific populations (breast and stomach groups).

All studies that contained information about BPM and/or CRRM in women with germline *CDH1* pathogenic or likely pathogenic variants (or mutations) were considered. We used the following electronic searches:

a. Breast: ((“Prophylactic Surgical Procedures”[Mesh] AND “Mastectomy”[Mesh]) OR (“Prophylactic Mastectomy”[Mesh] OR “prophylactic mastectomy”[Title/Abstract:~3] OR “prophylactic mastectomies”[Title/Abstract:~3] OR “prophylaxis mastectomy”[Title/Abstract:~3] OR “prophylaxis mastectomies”[Title/Abstract:~3] OR “risk reducing mastectomy”[Title/Abstract:~3] OR “risk reducing mastectomies”[Title/Abstract:~3] OR “risk reduction mastectomy”[Title/Abstract:~3] OR “risk reduction mastectomies”[Title/Abstract:~3])) AND (cdh OR “cdh1” OR “cdh‐1” OR cadherin OR “e‐cadherin” OR “ecadherin” OR “Cadherins”[Mesh] OR uvomorulin OR “Germ‐Line Mutation”[Mesh] OR “germ‐line*“ OR germline*);
b. Stomach: (((gastrectom* AND ptg) OR (Prophylactic total gastrectomy) OR (prophylactic gastrectomy) OR (risk reducing gastrectomy)) OR (“Prophylactic Surgical Procedures”[Mesh] AND “Gastrectomy”[Mesh])) AND (cdh OR “cdh1” OR “cdh‐1” OR cadherin OR “e‐cadherin” OR “ecadherin” OR “Cadherins”[Mesh] OR “Germ‐Line Mutation”[Mesh] OR “germ‐line*“ OR germline* OR “hereditary diffuse” OR “Genetic Predisposition to Disease”[Mesh] OR predisposition OR genetic).

Titles and abstracts were screened by two authors to determine whether studies met the eligibility criteria for full‐text assessment, and a sample of 25% was screened independently by another author as a quality assurance process. Full‐text assessment was conducted independently by two authors, with disagreements resolved by a third reviewer. Full‐text publications in English were considered. Congress abstracts were excluded.

We classified risk‐reducing surgeries as follows: BPM for healthy carriers, therapeutic mastectomy (TM) and CRRM in a woman who has been diagnosed with cancer in the other (ipsilateral) breast.

## 3 Results

A total of 11 studies were identified (between 2017 and 2025). These studies reported 352 individuals resulted positive at *CDH1* genetic testing. Among them, 234 (66.5%) were women, and 75 (32.1%) received any mastectomy (BPM or CRRM) (age ranged from 20 to 72 years). All risk‐reducing surgical procedures were performed in Western countries, in detail, five studies were from North America and six from Europe, respectively (Table 1).

**Table 1:** Overall summary of the studies identified in PubMed.

First Author	Year	Country	Women *CDH1* carriers	Mean age	Range	Treated women (%)	BPM	CRRM	Post‐operative histology
Feroce [12]	2017	Italy	4	40.5	30–64	1 (25)	—	1	—
Strong [13]	2017	USA	27	Unk	Unk	8 (29.6)	2	6	LCIS (1)
van der Kaaij [14]	2018	Netherlands	14	Unk	Unk	5 (35.7)	5	—	Unk
Klujit [15]	2018	Netherlands	20	45	20–72	2 (10)	2	—	LCIS (2)
Mirandola [16]	2019	Italy	7	44	Unk	1 (14.3)	1	—	LCIS (1)
Ruiz [17]	2019	Spain	3	Unk	Unk	1 (33.3)	—	1	Negative
Gianella [18]	2019	Spain	4	41.5	37–47	4 (100)	4	—	LCIS (2), ILC (1), negative (1)
Murphy [19]	2020	USA	9	Unk	Unk	9 (100)	3	6	Unk
Benesch [20]	2021	Canada	53	41.3	Unk	17 (32.1)	17	—	LCIS (2), ILC (1)
Boyaraz [21]	2024	USA	3	Unk	Unk	3 (100)	3	—	LCIS (3)
Famiglietti Gallanis [22]	2025	USA	90	Unk	27–37	24 (26.7)	15	9	Unk
**Total**			234			75	52	23	

*Note:* ‘*CDH1* carriers’ were women with positive *CDH1* genetic testing. ‘Treated women’ received any surgical procedures, bilateral prophylactic mastectomy or contra‐lateral risk reducing mastectomy. Number in brackets refer to the cases with positive or negative post‐operative histopathology.

Abbreviations: BPM, bilateral prophylactic mastectomy; CRRM, contralateral risk‐reducing mastectomy; LCIS, Lobular Carcinoma in situ; ILC, Invasive lobular carcinoma; Unk, Unknown data.

A total of 75 (32.1%) women were treated with BPM or CRRM, from a population of 234 resulted positive at *CDH1* genetic testing. In detail, 52 (69.3%) of women were treated with BPM and 23 (30.7%) with CRRM (women with diagnosed LBC before surgery) (Table 1). Articles about BPM or CRRM were reported only in Europe and North America; 61 (81.3%) of these women were treated in North America and 14 (18.7%) in Europe. In detail, 40 (76.9%) of BPMs were reported in North America, and 12 (23.1%) in Europe. Instead, 21 (91.3%) of CRRMs were reported in North America, and only 2 (8.7%) in Europe, respectively (Table 2).

**Table 2:** Geographic distribution of bilateral prophylactic mastectomy or contralateral risk‐reducing mastectomy performed in *CDH1* positive high‐risk women.

Country	Women *CDH1* carriers	Treated women (%)	BPM (%)	CRRM (%)
North America	182	61 (81.3)	40 (76.9)	21 (91.3)
Europe	52	14 (18.7)	12 (23.1)	2 (8.7)
**Total**	234	75	52	23

Abbreviations: BPM, bilateral prophylactic mastectomy; CRRM, contralateral risk‐reducing mastectomy.

The selected studies reported a positive family history for BC in 81.8% of the cases, and all were positive for LBC. A positive family history for DGC was also identified in 72.7% (Table 3).

**Table 3:** Reported studies associated with family history information in *CDH1* women who received at least one mastectomy.

First Author	Family BC history	Family LBC history	Family GC history
Feroce [12]	Positive	Positive	Positive
Strong [13]	Positive	Unk	Positive
van der Kaaij [14]	Positive	Positive	Positive
Klujit [15]	Positive	Positive	Positive
Mirandola [16]	Positive	Positive	Positive
Ruiz [17]	Positive	Unk	Positive
Gianella [18]	Positive	Positive	Positive
Murphy [19]	Unk	Positive	Unk
Benesch [20]	Unk	Unk	Unk
Boyaraz [21]	Positive	Unk	Negative
Famiglietti Gallanis [22]	Positive	Unk	Positive

Abbreviations: BC, Breast Cancer; LBC, Lobular Breast Cancer; GC, Gastric Cancer; Unk, Unknown data.

Except for two cases resulted that resulted in negative, all post‐operative histopathological analysis revealed in all BPM or CRRM specimens' lobular carcinoma *in situ* (LCIS), and invasive LBC was reported also in two cases (Table 1).

## 4 Discussion

Our research demonstrated that all studies were from North America (USA and Canada) and Europe (Italy, Netherlands and Spain). Although only eleven studies were identified, these data resulted in line with the geographic *CDH1* variants distribution. This evidence reflects the high heterogeneity of germline *CDH1* variants distribution in the world, with significant variations between areas with different incidence rates of GC. Targeted and highest frequency of hot‐point germline *CDH1* variants are identified in countries in which GC risk is notoriously low, for example, USA, Canada, North Europe [23]. We observed, in fact, that BPM or CRRM surgeries are reported only in GC low‐risk countries in which a high proportion of germline *CDH1* variants are routinely detected in high‐specialized clinical genetic centers.

Regarding the frequency of risk‐reducing procedures, less than half (75/234) of the screened women included in these studies were treated with a BPM or CRRM. Literature reported ‘only’ 75 BPM or CRRM in women carrying germline *CDH1* pathogenic or likely pathogenic variants.

If we compare risk‐reducing procedures in women *BRCA1/2* carriers, we observe that the overall frequency is greatly different. A recent systematic review and metanalysis of 21 studies on women *BRCA1/2* carriers, reported more than 12,300 women treated with BPMs or risk‐reducing mastectomies, in a sample size of more 50,000 individuals *BRCA1/2* carriers [24].

No systematic studies are available about the frequency of mastectomies performed in women carrying germline non‐*BRCA* genes pathogenic or likely pathogenic variants, classified recently as high/moderate risk for BC development (i.e. *TP53, PALB2, PTEN, CHEK2, ATM, CDH1*, etc.). It would be very interesting to evaluate this data to compare the different frequencies among non‐*BRCA* genes.

The decision‐making process for a BPM or CRRM differs significantly between *CDH1* and *BRCA1/2* gene mutation carriers, due to differences in histological tumor sub‐types, co‐occurring organ cancer risks, and screening approaches. While both are high‐penetrance genes that warrant serious discussion about risk‐reducing surgery, their clinical pathways diverge greatly. For example, *BRCA1/2* are associated mostly with ductal carcinomas. Instead, *CDH1* explicitly linked to invasive lobular carcinoma. Also, *CDH1* carriers are at high‐risk for diffuse gastric cancer predisposition.

Although the frequency of risk‐reducing mastectomies performed in *CDH1* carriers is very low, a consideration about the overall frequency of *CDH1* variants should be considered.

In LBC, the overall frequency of germline *CDH1* variants is very low, lower than DGC. If we consider the so‐called HLBC syndrome, in which LBC is the first clinical manifestation, the frequency of these variants is around 1%–3% [5].

We should also consider that prophylactic or risk‐reducing mastectomies are routinely performed in *CDH1* high‐risk women, and probably mostly of these data are not all published. However, it remains unclear whether the lower uptake reflects mutation rarity, the differences in BC risk (penetrance) compared to *BRCA1/2*, or the variations in clinical decision‐making.

Exploring family history, all *CDH1* women considered eligible for BPM or CRRM, presented a positive family history for BC, specifically for LBCs. Family history has significantly contributed to understanding inherited diseases, familial and hereditary cancer syndromes in particular. To assess the cancer risk for unaffected family members and to identify a possible genetic cause, it is important to describe a detailed family history, including information about life habits, gender, age at onset, affected members, and the number of generations. Therefore, a careful anamnesis focused on oncological data lead to the diagnosis of familial and/or hereditary cancer. In *CDH1* carriers, a positive family history remains one of the most important risk factors for developing cancer. Roberts et al., in fact, described a lower GC risk in a sporadic setting in individuals CDH1 carriers, in comparison to those with a positive family history [8]. Our evidence supports that a positive family history for BC is frequently associated with the performed risk‐reducing surgeries in high‐risk individuals, and BPM or CRRM should be considered with caution in women with a sporadic BC manifestation, as well as those carrying variants of unknown significance.

Interestingly, except for one, all post‐operative breast specimens after histopathological examination revealed multiple foci of LCIS in BPM or CRRM (Table 2). In a sporadic setting, LCIS is considered a risk factor that does not always lead to invasive BC. While the historical approach has shifted from radical mastectomy, LCIS is still considered a marker of increased risk for developing invasive cancer, with an approximate 9‐ to 10‐times higher risk than the general population [25]. About the hereditary setting, in particular in high‐risk individuals with a documented germline *CDH1* pathogenic or likely pathogenic gene, there is very little in the literature. In cases of bilateral LCIS/invasive LBC within the GLACIER study, 4 of 50 harbored germline *CDH1* mutations, with 3 truncating mutations and 1 splice site mutation. If women under 45 were considered, 28% of patients with bilateral LCIS had underlying germline mutations. A literature review of 408 cases of LCIS/ILBC with no history of HDGC revealed only three germline *CDH1* mutations [4]. This included one series of 65 cases of LCIS, 17 of which were bilateral, in which no germline *CDH1* mutations were identified [26].

Finally, our scoping review presented some important limitations. Due to the rarity of this inherited cancer predisposition and to the low number of surgical procedures reported in PubMed database we were not able to perform a complete statistical analysis including a metanalysis and heterogeneity of studies evaluation Also, the risk bias evaluation was not assessed. For this reason, we decided to approach at this research as a scoping review. Other limitation is related to a possible publication bias. Only Western studies are reported, with a limited data availability of unpublished surgical procedures.

## 5 Conclusions

In this review, we observed that BPM or CRRM is performed in about 1/3 of women with germline *CDH1* variants and reported only in Western countries, in accord with published data. A positive family history for LBC, is frequently associated with the reported breast risk‐reducing surgeries. Moreover, in post‐operative specimen's breast tissues, LCIS was identified frequently.

## Author Contributions

Study design: G.C.; Literature research: G.C., A.P., E.B., A.D.S., S.D.S.; Data collection: G.C., E.M.; Data extraction: G.C., C.L.V.; Draft of the manuscript: G.C., C.L.V.; Critical revision: F.D.L., M.I., P.V., V.G. All authors have read and commented on the working versions and approved the final manuscript before submission.

## Synopsis

- Germline *CDH1* pathogenic or likely pathogenic variants confer an increased risk for lobular breast carcinoma development. Risk‐reducing mastectomy is considered a possible strategy to mitigate the risk in women with *CDH1* mutation.
- Literature reports very few articles about bilateral prophylactic mastectomy or contralateral risk‐reducing mastectomy performed in *CDH1* mutation carriers. Articles are reported only in Europe and North America.
- Around 1/3 of women with germline *CDH1* pathogenic or likely pathogenic variants are treated with risk‐reducing surgery, and the majority (over 80%) present a positive family history for breast carcinoma and foci of lobular tumor in post‐operative breast specimens.

## Data Availability

The data that support the findings of this study are available on request from the corresponding author. The data are not publicly available due to privacy or ethical restrictions.
